# HslO ameliorates arrested *ΔrecA polA* cell growth and reduces DNA damage and oxidative stress responses

**DOI:** 10.1038/s41598-022-26703-z

**Published:** 2022-12-23

**Authors:** A. Kaidow, N. Ishii, S. Suzuki, T. Shiina, K. Endoh, Y. Murakami, H. Kasahara

**Affiliations:** 1 Department of Biology, School of Biological Sciences, Tokai University, Sapporo, 005-8601 Japan; 2 Department of Molecular Medicine, School of Medicine, Tokai University, Isehara, 259-1193 Japan; 3 Hokkaido Regional Research Center, Tokai University, Sapporo, 005-8601 Japan

**Keywords:** Genetics, Microbiology, Molecular biology

## Abstract

Chromosome damage combined with defective recombinase activity has been widely considered to render cells inviable, owing to deficient double-strand break repair. However, temperature-sensitive *recAts polA* cells grow well upon induction of DNA damage and supplementation with catalase at restrictive temperatures. These treatments reduce intracellular reactive oxygen species (ROS) levels, which suggests that *recAts polA* cells are susceptible to ROS, but not chronic chromosome damage. Therefore, we investigated whether *polA* cells can tolerate a complete lack of recombinase function. We introduced a *ΔrecA* allele in *polA* cells in the presence or absence of the *hslO*-encoding redox molecular chaperon Hsp33 expression plasmid. Induction of the *hslO* gene with IPTG resulted in increased cell viability in *ΔrecA polA* cells with the *hslO* expression plasmid. *ΔrecA polA* cells in the absence of the *hslO* expression plasmid showed rich medium sensitivity with increasing ROS levels. Adding catalase to the culture medium considerably rescued growth arrest and decreased ROS. These results suggest that *hslO* expression manages oxidative stress to an acceptable level in cells with oxidative damage and rescues cell growth. Overall, ROS may regulate several processes, from damage response to cell division, via ROS-sensitive cell metabolism.

## Introduction

As most bacteria have only one chromosome, cell division is delayed by the induction of transcription when DNA damage is sensed; this phenomenon is called the SOS response^1^. Defects in DNA polymerase I (pol I) function, such as the *polA25* mutation, cause DNA damage via nick and gap accumulation from failures when processing Okazaki fragments. When a replication fork encounters a discontinuity in a DNA template, a double-strand break (DSB) occurs in *Escherichia coli* chromosomes. The *E. coli* RecA protein plays crucial roles in homologous recombination and repair^2^, functioning both as a recombinase and coprotease. Recombination is accompanied by an extensive DNA replication process called recombination-dependent DNA replication (RDR) which repairs collapsed replication forks^3^.

Synthetic lethality, in which the combined knockout of two nonessential genes is lethal, has direct applications in understanding cellular processes. *RecA polA* and *recB polA* double mutants are inviable owing to deficiencies in DSB repair^4^. The LexA protein, an SOS repressor, regulates SOS gene expression in response to DNA damage. *LexA71* mutations completely inactivate the LexA repressor, which de-represses the LexA regulon^5^. Recently, we reported that *recAts polA* cells with *lexA* mutations become temperature-resistant in the presence of the *hslO* gene, a member of the heat shock locus genes, which encode a redox molecular chaperone^6^. Further, temperature sensitivity is suppressed by catalase, which is related to reactive oxygen species (ROS) degradation. This finding indicates that temperature sensitivity is synchronised to intracellular ROS levels rather than chromosome degradation. *hslO*, which encodes Hsp33 (HslO), is a redox molecular chaperone that protects organisms against oxidative stress that leads to protein unfolding^7^. Loss of *hslO* function sensitises cells to hydrogen peroxide^8^. HslO activation is triggered by the oxidative unfolding of its redox-sensor domain^9^, which classifies HslO as a member of recently discovered chaperones that require partial unfolding for full activity^10^.

Recent studies have highlighted the contribution of stress-stimulated ROS accumulation^11^, which induces cell death^12^. However, ROS accumulation appears to be bacteriostatic rather than bactericidal^13^. Thymine starvation leads to the accumulation of both single-strand DNA regions and intracellular ROS^14^. Hong et al. also reported that lethality was induced by stimulating self-amplifying ROS accumulation that overwhelmed primary damage repair^12^. Cells possessing genetic mutations related to recombination repairs, such as *priA* and *recB*, are known to be sensitive to rich mediums. Furthermore, cells face replicative stress during growth^15^. However, ROS, like nitric oxide (NO) in the nervous system, have a short half-life owing to their high reactivity. Therefore, ROS could function as effectors because of high reactivity and may serve as stress markers^16^ and signalling factors^17^.

The relationship between DNA damage, cell proliferation, and ROS production has remained unclear. In this study, we explored the growth of *ΔrecA polA* cells in the presence of ROS to elucidate the pathways involved. By investigating ROS as a determinant for cell growth, the intricacies of redox signalling in *E. coli* can be further understood.

## Results

### *ΔrecA polA* cells grow well with *hslO* expression plasmid

We examined whether *hslO* expression was sufficient to suppress *recA200* (Ts) *polA25 lexA*^+^ cell (AQ10549) sensitivity to temperature. A *polA25* mutation was inserted in the polymerase domain of the *polA* gene resulting in a loss of polymerase activity. Plasmids used for complementation tests were transformed into AQ10549 cells. The temperature sensitivity of the transformed cells was confirmed by colony formation assay. Cells with *p*SrpC (which express *hslO*) and *p*SRO*ΔyrfG* (which express *hslR* and *hslO*) were viable at 42 °C, in contrast with those transformed with empty vector plasmid (Supplementary Fig. S1). This result suggests that *hslO* expression alone is partially sufficient to suppress lethality. Conversely, pSRO1 failed to suppress lethality. This suggests that the upstream region of *yrfG* can possess a negative effect for *recA polA* lethality in *lexA*^+^ cells. Thus, *p*SrpC could improve *recA* (Ts) *polA* cell viability at 42 °C.

**Figure 1:**
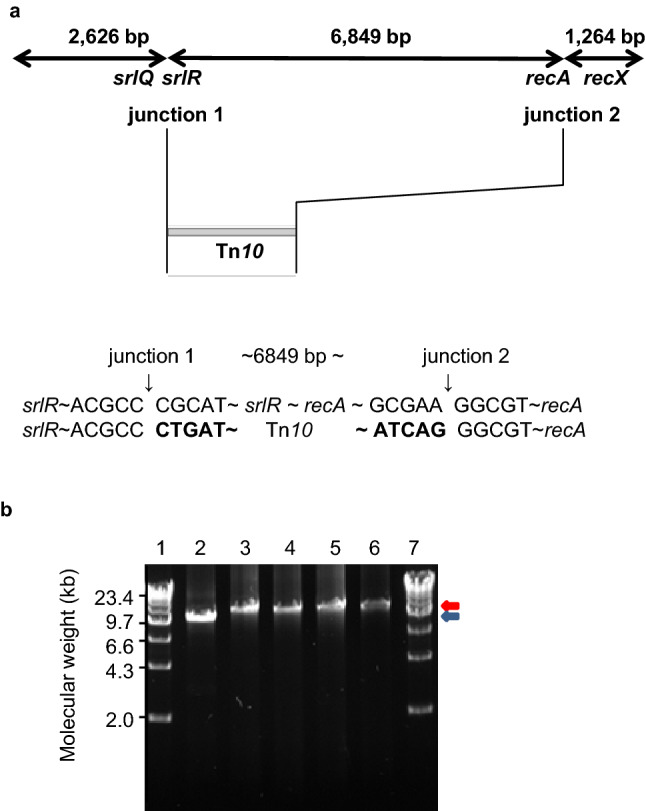
Tolerance of *polA25* cells for introducing a *ΔrecA306* allele with P1 transduction. (**a**) Genome sequence reference mapping of AQ10459 and AQ10870 against the MG1655 genome and junction points. In (**a**), the top line indicates that the DNA region matches AQ10459 and AQ10870; left 2643 bp and right 1264 bp, or miss in AQ10870; centre 6849 bp with the gene location. Junction sequences of AQ10459 and AQ10870 are shown in the centre and bottom lines, respectively. The results of reference mapping are shown. (**b**) Analysis of *recA* alleles in *ΔrecA polA* candidate cells using long PCR. Long PCR was carried out as described in “Methods”. Results of 0.8% agarose gel electrophoresis are shown. Lane 1: marker, lane 2: AQ10459, lane 3: AQ10870, lane 4: *ΔrecA306* derivative, lane 5: TK1230 cells, lane 6: TK1224 cells, lane 7: marker. Arrow heads indicate the long PCR product from *recA*^+^ (blue) or *ΔrecA306* (red). The gel image in (**b**) shows a flipped image of the appropriate area without either the extra unnecessary markers or the gel origins from the original photograph (Fig. S2).

Next, we attempted to construct *ΔrecA polA* cells, because molecular chaperones, such as HslO, might have restored the temperature sensitivity of the recombinase activity of RecA(Ts). AQ11756 (*polA25*) cells were transformed with either empty vector or *lacZ* leader fusion *hslO*-expressing plasmids (*p*EX*srpC*, which is a derivative of *p*MW119). These cells were infected with P1 lysates from AQ11756 cells to transduce a *ΔrecA306* allele. We selected tetracycline resistant (Tc^r^) colonies using IPTG, which induced *hslO* expression in *p*EX*srpC*-containing cells. We observed 2 or > 20 *ΔrecA* candidates in 10^4^ Tc^r^ colonies from AQ11756 cells containing an empty vector or *p*EX*srpC*, respectively. We then carefully analysed those candidates because the induced mutation was believed to be lethal. Thus, we verified the absence of the *recA* allele using colony PCR. We examined the presence of the *ΔrecA306* allele in the candidates using long PCR products: *recA*^+^ and *ΔrecA306* (10.7 and 13 kb, respectively) (Fig. 1, Fig. S2). All the candidates possessed *ΔrecA306* alleles. We further verified the loss of the *recA* gene from the candidates using next-generation sequencing (NGS), as described previously^18^.

**Figure 2:**
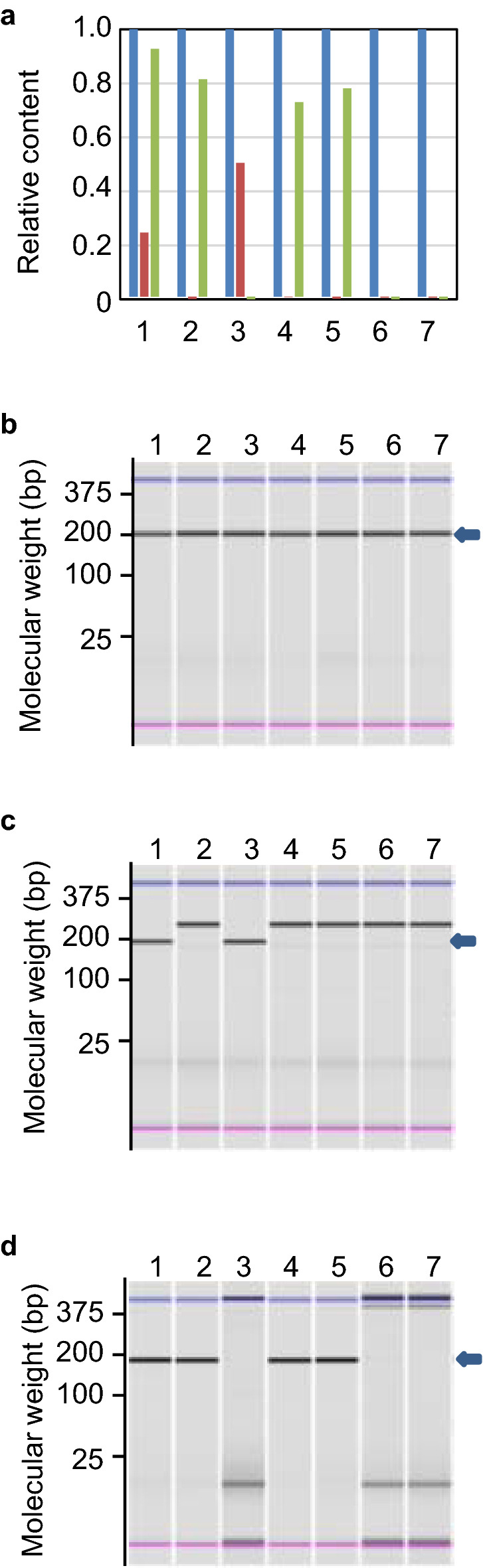
Confirmation of *ΔrecA306 polA25* candidate cells using qPCR. (**a**) Relative ratio of the polymerase domain of *polA* and *recA* against the 5′–3′ exonuclease domain of *polA* in TK1224 and TK1230 cells. Chromosomal DNA was prepared from the indicated cells using a CellEase Bacteria II kit, and 2 μL of lysed cell solution was analysed for absolute amounts of target DNA. The relative ratios of the polymerase domain of *polA* and *recA* were calculated against the 5′–3′ exonuclease domain of *polA*. The blue bar corresponds to the ratio of the 5′–3′ exonuclease domain of *polA*. The red and green bars indicate the relative ratios of the polymerase domain of *polA* and *recA*, respectively. Analysed samples were lane 1: AQ10459, lane 2: AQ11756, lane 3: AQ10870, lane 4: AQ1217, lane 5: TK1219, lane 6: TK1230, lane 7: TK1224. (**b**–**d**) Confirmation of amplified 5′–3′ exonuclease domain, polymerase domain, and products of *recA*, respectively, of *polA* using ChIP electrophoresis. The qPCR products were analysed using ChIP electrophoresis. The arrowheads indicate either amplified 5′–3′ exonuclease (**b**), polymerase (**c**) or *recA* fragments (**d**). The analysed samples were in the same order as in (a). The original gel images (Fig. S3) were cut for suitability and ease of comparison.

The NGS results of the *recA* region confirmed that purified Tc^r^ clones possessed *ΔrecA306* sequences. Subsequently, we quantified absolute DNA amounts for the 5′–3′ exonuclease and polymerase domains of the *polA* and *recA* genes using qPCR. The ratios of the 5′–3′ exonuclease and polymerase domains of the *polA* and *recA* genes are shown in Fig. 2a. These results indicate that the candidates of *ΔrecA polA* cells, TK1230 and TK1224 cells, did not possess the polymerase domain of *polA* nor *recA* alleles. These qPCR products were further analysed by ChIP electrophoresis (Fig. 2b–d, Fig. S3). The results indicated that the PCR products possessed proper amplicon size. Thus, we could construct *ΔrecA306 polA* cells, which were presumed to be inviable, using both empty vector and pEX*srpC* plasmids (TK1230 and TK1224), respectively.

**Figure 3:**
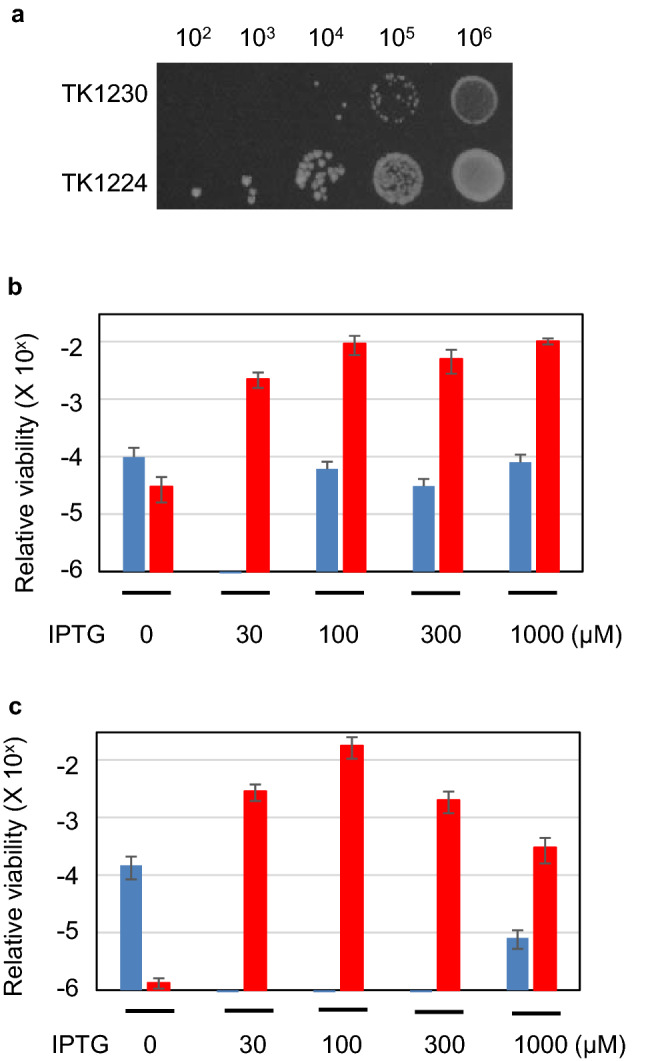
Effect of IPTG on *ΔrecA306 polA25* cell viability. (**a**) Colony formation by *ΔrecA306 polA25* cells. TK1230 (*ΔrecA306 polA p*vec) or TK1224 (*ΔrecA306 polA p*EXsrpC) cells were grown in M9GCAA medium and subsequently diluted with M9B to contain the indicated number of cell particles of either TK1230 or TK1224 cells. Next, 2 μL of diluted cells was spotted on LA plates and incubated at 30 °C for 3 days. (**b**) Effect of IPTG on relative viabilities of *ΔrecA306 polA25* cells. Relative viabilities (RV) of TK1230 and TK1224 cells are shown with a bar graph. Cells were grown in M9GCAA medium. IPTG concentrations in the plates are indicated below. Cells were diluted with M9B from 10^4^ to 10^7^ cell particles/mL. Diluted cell cultures were spread on M9GCAA plates supplemented with various IPTG concentrations (indicated below). Viable cell counts were determined 5 days after cultivation at 30 °C. The results are shown as the mean ± SEM (n ≥ 3). TK1230: blue, TK1224: red. (**c**) Effect of IPTG on relative viabilities of *ΔrecA306 polA25* cells from 30 μM IPTG seed culture. RVs of TK1230 and 1224 cells grown with 30 μM IPTG are shown with various concentrations of IPTG plates in the same manner as (**b**).

Colony formation of both TK1230 (*ΔrecA polA25 p*vec) and TK1224 (*ΔrecA polA25 p*EX*srpC*) cells is shown in Fig. 3a. TK1224 cells showed 10-fold higher viability compared to TK1230 cells on L plates. Then, colony-forming abilities against particles of TK1224 (*ΔrecA polA25 p*EX*srpC*) and TK1230 (*ΔrecA polA25 p*vec) cells were determined both in seed cultures and plate medium (Fig. 3b,c). In the absence of IPTG in seed culture, TK1230 cells were viable. However, the relative viability was less than 10^−4^ for all plates under IPTG conditions (Fig. 3b). In contrast, TK1224 cells responded to several plates containing various IPTG concentrations, although the relative ratio of TK1224 in the absence of IPTG was as low as TK1230 cells (Fig. 3b). In this experiment, maximum viability was observed in seed culture with 30 μM IPTG; we demonstrated the effects of IPTG concentration on the relative viability with 30 μM IPTG seed culture (Fig. 3c). The relative viability of TK1224 at 100 μM IPTG was 100-fold greater than that of TK1230 cells. These data showed that *hslO* expression alleviated the lethality of *ΔrecA polA* cells (Fig. 3b,c). This result agreed with the result in Fig. 3a. Thus, *hslO*-expressing plasmids support *ΔrecA polA* cell viability while inducting *hslO* expression. Therefore, *hslO* expression affects *ΔrecA polA* cell viability similar to *recAts polA* cells, as reported previously^6^.

### *ΔrecA polA* cells are sensitive to rich medium

Essential cellular processes are investigated by using conditional mutants. An advantage of *recAts polA* cells is their temperature sensitivity, which enabled us to investigate why these cells failed to grow. In our experiment using *ΔrecA polA* cells, we found that *ΔrecA polA* cells exhibited sensitivity to L medium.

In liquid medium, TK1230 (*ΔrecA polA25 p*vec) cells grew very slowly or poorly in the M9GCAA medium and failed to grow in the L medium (Supplementary Fig. S4). However, TK1224 (*ΔrecA polA25* pEX*srpC*) cells grew in all tested media. The results might explain the difficult TK1230 strain construction described above. TK1230 and TK1224 cells were mixed with soft agar to test whether the cells possessed a density-dependent growth phenotype in solid medium. TK1230 cells failed to grow on L plates, in contrast to those on M9GCAA plates. TK1224 cells grew on both M9GCAA and L plates (Supplementary Fig. S4). Thus, TK1230 cells are sensitive to rich medium, i.e., a rich medium-sensitive phenotype.

**Figure 4:**
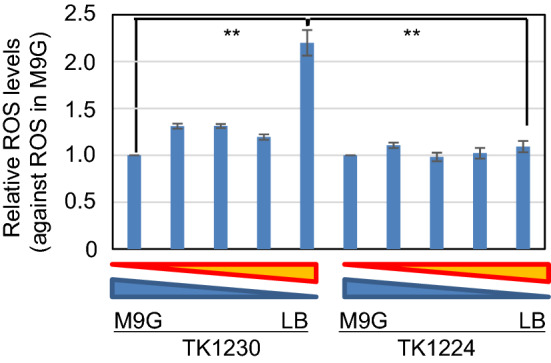
Effect of culture medium on ROS levels in *ΔrecA306 polA25* cells. Fully grown TK1230 or TK1224 cells were inoculated in various media at 2 × 10^7^ particles/mL (approximately 0.01 OD_600_) and cultured for 24 h at 30 °C. ROS levels were analysed as in the “Methods”. From left to right, M9GCAA, 25% L medium with M9GCAA, 50% L medium with M9GCAA, 75% L medium with M9GCAA, and L medium. The results are shown as the mean ± SEM (n ≥ 3). Double asterisks indicate significant differences (*p* < 0.01) as analysed using Welch’s *t* tests (n = 14).

ROS levels in TK1230 and TK1224 cells were measured at various medium compositions, and we demonstrated the relative ROS levels of cells grown in M9GCCA medium with various medium compositions (Fig. 4). The results showed that TK1230 cells cultivated in L medium had significantly increased relative ROS levels compared with those cultured in other conditions (*p* < 0.01, Welch’s *t* test). This finding indicates that the decrease in growth is synchronised to the increase in ROS levels, with regard to the medium composition.

### Growth and ROS levels appear to be inversely correlated

**Figure 5:**
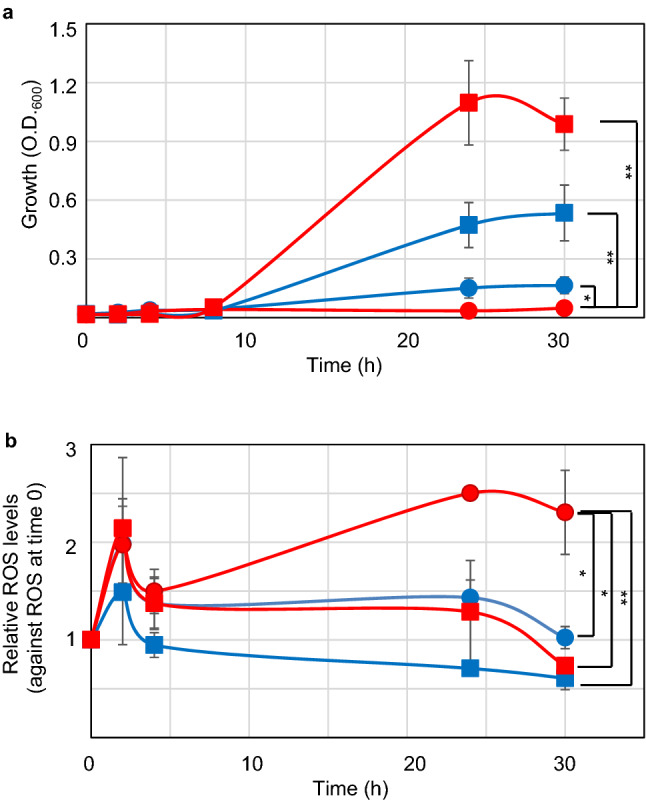
Effect of culture medium on growth and ROS levels in *ΔrecA306 polA25* cells. (**a**) Effect of medium on *ΔrecA306 polA25* cell growth. Fully grown TK1230 or TK1224 cells were inoculated in either M9GCAA or LB medium at 2 × 10^7^ particles/mL (approximately 0.01 OD_600_). Cell growth was measured using OD_600_ at the indicated times. TK1230 cells (round) and TK1224 cells (square) in M9GCAA (blue) or L medium (red) are shown. The results are shown as means ± SEM (n ≥ 4). OD_600_ at 30 h were analysed using Welch’s *t* tests (n = 7). Asterisks and double asterisks indicate *p* < 0.05 and *p* < 0.01, respectively. (**b**) Effect of culture medium on ROS levels of *ΔrecA306 polA25* cells. Fully grown TK1230 or TK1224 cells were inoculated in either M9GCAA or L medium at 2 × 10^7^ particles/mL (approximately 0.01 OD_600_). Average ROS levels (RFU) were measured at the indicated times. Symbols are the same as those described in (**a**). The results are shown as the mean ± SEM (n ≥ 4). Relative ROS levels at 30 h were analysed using Welch’s *t* tests (n = 7). Asterisks and double asterisks indicate *p* < 0.05 and *p* < 0.01, respectively.

AQ1230 (*ΔrecA polA p*vec) and TK1224 (*ΔrecA polA p*EXsrpC) cells were inoculated at 2 × 10^7^ cell particles/mL in M9GCAA or L medium at 30 °C. Samples were collected from the cultures and analysed for optical density (OD_600_) (Fig. 5a) and relative ROS levels (Fig. 5b). Both TK1230 and TK1224 cells did not grow until 8 h after inoculation. TK1224 cells reached almost full growth at 24 h after inoculation in both M9GCAA and L mediums. TK1230 cells grew very slowly in M9GCAA but failed significantly to grow in L medium until 30 h (p < 0.05, Welch’s *t* test) (Fig. 5a). Intracellular ROS accumulation was observed at 2 h after inoculation in both cultivation conditions in TK1230 and TK1224 cells. Then, the ROS levels decreased 4 h after inoculation and remained low until 30 h, except for in TK1230 cells cultured in L medium (Fig. 5b). Interestingly, TK1230 cells cultured in L medium had significantly increased intracellular ROS levels at 30 h (p < 0.05, Welch’s *t* test), while the transiently increased ROS levels in both TK1230 and TK1224 cells could result from respiration and macromolecule synthesis during the lag phase. Therefore, growth failure did not result from transiently increased ROS levels at the beginning of culture, but rather from the increased ROS levels. Thus, relatively high ROS levels (> 2-folds) was synchronised with their loss of growth in TK1230 cells in liquid medium.

**Figure 6:**
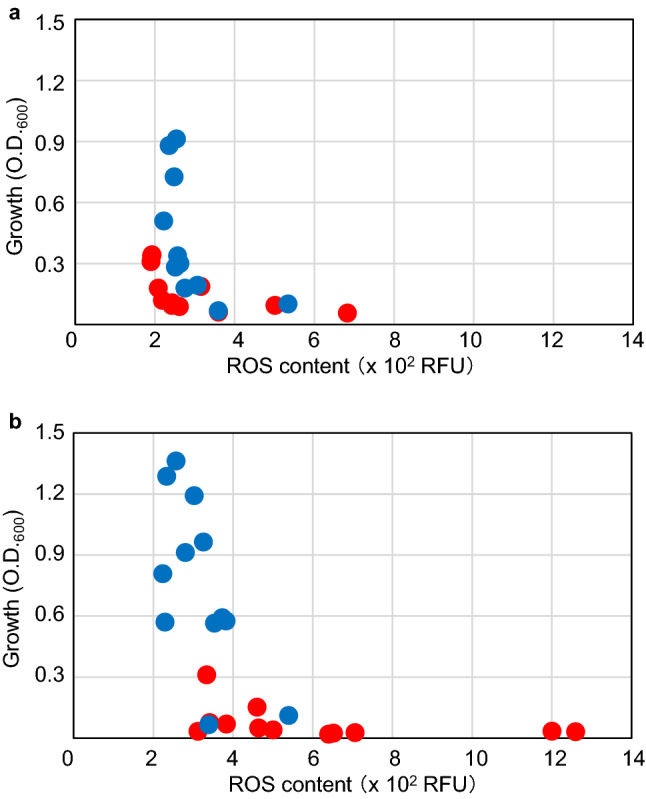
Analysis of growth and ROS levels in *ΔrecA306 polA25* cells. (**a**) Sensitivity of *ΔrecA306 polA25* cells to M9GCAA medium. For TK1230 and TK1224 cell samples grown in M9GCAA medium, two-dimensional scatter plots were constructed with the mean ROS levels and absorbance on the x and y axes, respectively. TK1230 and TK1224 are indicated using red and blue, respectively. (**b**) Sensitivity of *ΔrecA306 polA25* cells to L medium. For TK1230 and TK1224 cell samples grown in L medium, two-dimensional scatter plots were constructed as described in (**a**).

We plotted the data from 24 h after inoculation in scatter plots with ROS levels on the x-axis and growth on the y-axis. The results for M9GCAA and L medium are shown in Fig. 6a,b, respectively. TK1230 cells showed an L-figured distribution that aligned with 200 RFU or 0.1 abs (OD_600_) (Fig. 6a), while TK1224 cells were mainly aligned at 250 RFU with some overlap with TK1230 cells. TK1230 cells were aligned at 0.05 abs in L medium (Fig. 6b). However, TK1224 cells were broadly distributed in these growth and ROS levels and showed less overlap with those of TK1230 cells, between 0.6 to 1.4 abs and 200 to 400 RFU, respectively. Thus, in contrast to those of TK1224 cells, TK1230 cells failed to grow with increasing ROS levels. Therefore, growth failure in a rich medium could correlate with ROS accumulation, while it seemed that TK1224 cells had less increasing ROS levels and were also less responsive to elevated ROS levels. This result might suggest that TK1224 cells have a relatively increased threshold for ROS levels (Fig. 6a). It was interesting that TK1230 cells had higher ROS levels in the L medium than on M9GCAA, suggesting that intracellular ROS levels were involved in arresting the growth of *ΔrecA polA* cells, similar to previous observations in r*ecAts polA* cells^6^. Thus, our results suggest that *hslO* expression ameliorates ROS levels.

### Addition of catalase stimulates colony formation of *ΔrecA polA* cells

In Fig. 6, the severe growth deficiency was synchronized with the ROS levels. Thus far, our results may indicate that intracellular ROS levels play an important role in reducing *ΔrecA polA* cell growth. In a previous study^6^, we showed that adding catalase to the culture medium enabled *recAts polA* cells to grow at restrictive temperatures. Hydrogen peroxide (H_2_O_2_) can pass through cell membranes and get converted into hydroxy radicals. The addition of catalase to the medium will result in decreased H_2_O_2_ concentrations in surrounding cells. Even if the accumulated H_2_O_2_ in those cells is harmful, cells may be able to continue growing upon the addition of catalase to the medium, reducing the H_2_O_2_ in the medium and decreasing their ROS levels below the postulated threshold levels by transmission equilibrium. This possibility coincides with a mirror image of the autocrine and/or paracrine growth factor depletion experiments in animal cells. Therefore, we investigated the effect of catalase on rich medium-sensitive *ΔrecA polA* cells.

**Figure 7:**
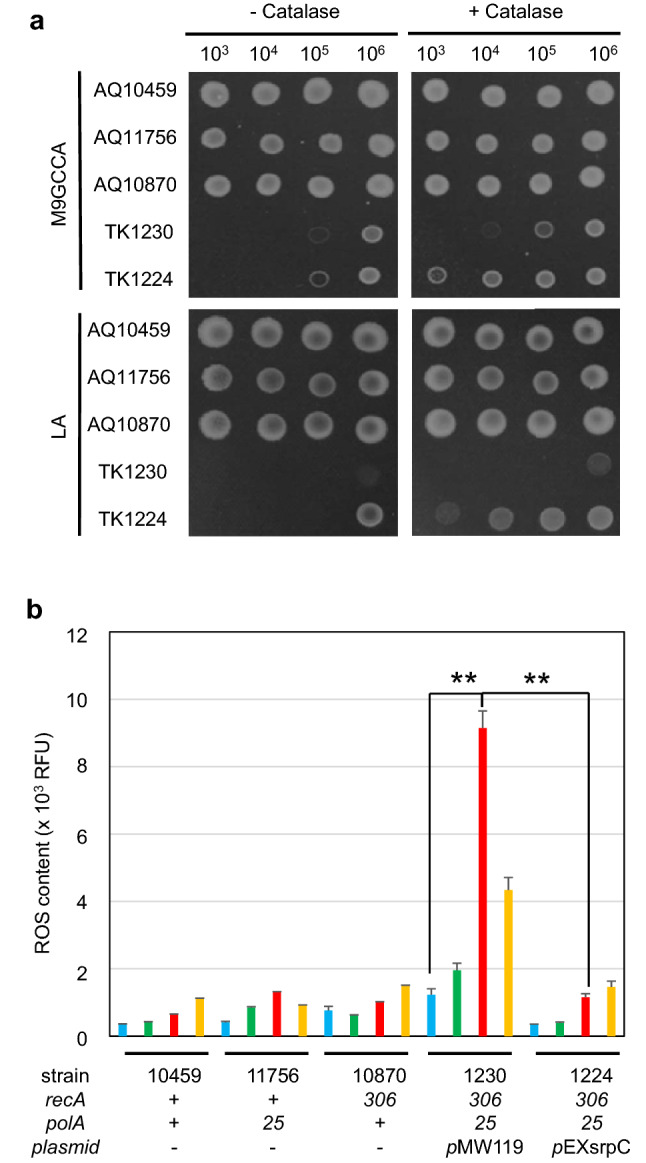
Effect of catalase on L medium sensitivity in *ΔrecA306 polA25* cells. (**a**) Effect of catalase in *ΔrecA306 polA25* cells growing on plate medium. TK1230 (*ΔrecA306 polA p*vec) or TK1224 (*ΔrecA306 polA p*EXsrpC) cells were fully grown in M9GCAA medium and subsequently diluted with M9B to contain the indicated number of cell particles. Then, 2 μL of diluted cells were spotted on L plates and incubated at 30 °C for 30 h. The images are shown from top to bottom: M9GCAA and L plate medium; right to left: no catalase and 1000 U/mL, respectively, at 30 °C. (**b**) Effect of catalase on ROS accumulation in *ΔrecA polA* cells. The cells shown in (**a**) were recovered and stained with SYBR Green I and CellRox Deep Red. Nucleic acid-containing *E. coli* particles were selected from the double-stained samples and analysed using histograms. Each histogram was as follows: blue, M9GCAA without catalase; green, M9GCAA with catalase; red, L without catalase; orange, L with catalase. Each bar represents the mean ± SEM (n ≥ 4). Double asterisks indicate significant differences (*p* < 0.01) as analysed using Welch’s *t* tests (n = 6).

TK1230 cells grew on M9GCAA plates with only 10^6^ cell particles but failed completely to grow on L plates at 30 °C (Fig. 7a), while TK1224 cells grew with 10^6^ cell particles on both M9GCAA and L plates. Thus, TK1230 cells showed an L medium-sensitive phenotype. We investigated the effect of catalase on TK1230 and TK1224 cell growth on M9GCAA and L plates. TK1230 cells showed slight stimulation of colony formation on M9GCAA and L medium upon addition of catalase. Interestingly, TK1224 cells formed colonies with catalase, even with as few as 10^3^ cells per spot. Thus, catalase supplementation enabled TK1224 cells to form colonies approximately 100-fold more efficiently than TK1230 cells on both M9GCAA and L plates. These results show that *hslO* plasmid enables *ΔrecA polA* cells to grow well on plates with L medium, suggesting that *hslO* alleviates the rich medium-sensitive phenotype, i.e. rich medium stress (Fig. 7a, lower panels). Simultaneously, reduced colony formation by *ΔrecA polA* cells was ameliorated with catalase treatment, especially in TK1224 cells, indicating that *ΔrecA polA* cells are viable. This effect is likely caused by the detrimental effects of hydrogen peroxide because it is ameliorated by catalase (Fig. 7a, right). Further, *hslO* plasmid enables *ΔrecA polA* cells to grow well on plate medium with catalase treatment, suggesting that *hslO* gene expression is pivotal for *ΔrecA polA* cell growth. However, TK1224 cell colony formation did not have levels observed in isogenic cells, including wildtype (AQ10459), *polA* (AQ11756), and *ΔrecA* (AQ10870) cells. These results suggest that essential problem(s) in *ΔrecA polA* cells were not restored completely with *hslO* expression.

We measured ROS levels in these colonies to assess the effect of intracellular ROS. We observed that ROS levels in wild, *polA*, and *ΔrecA* cells were less than 2000 RFU, suggesting that these cells on plate culture can grow with ROS levels around 2000 RFU (Fig. 7b). Additionally, ROS levels observed in cells cultured in the L medium were relatively higher than those in the M9GCAA medium, suggesting that ROS levels are increased by cell growth. The increased ROS levels may arise from the respiratory chain and metabolic decomposition products during cell growth. ROS levels from TK1230 cells cultured on M9GCAA plates or TK1224 cells cultured on M9GCAA or L plates satisfied the hypothetical ROS levels above, i.e., < 2000 RFU, which enabled colony formation. The data also indicate that simultaneous deficiency of RecA and Pol I caused TK1230 cells to accumulate high ROS levels in comparison with those of AQ10459, AQ11756, and AQ10870 cells. TK1224 cells from L plates had ROS at around 2000 RFU and showed considerable growth on L plates either with or without catalase, especially with stimulation of colony formation by catalase. In contrast, TK1230 cells failed to form colonies without catalase, showing ROS levels of > 9000 RFU. When TK1230 cells were supplemented with catalase, the ROS levels decreased to less than 4000 RFU and the cells could form colonies but very poorly. TK1230 cells on the L plate with catalase formed very small colonies that had lower ROS levels, indicating that ROS levels, presumably from hydrogen peroxide and its derivatives, again determine whether *ΔrecA polA* cells can form colonies. This estimation was supported by the fact that ROS levels in TK1230 cells cultured on L plates without catalase were significantly different from cells cultured on M9GCAA plates and those of TK1224 cells cultured on L plates (p < 0.01, Welch’s *t* test). These results indicate that *polA25* mutant cells were tolerant to completely absent RecA function. Additionally, our results show the importance of ROS degradation for cell growth, suggesting that ROS is one of the determinants of cellular growth in injured cells.

## Discussion

Previously, we investigated *recAts polA* cells with a suppression of lethality (Srp) mutation that suppresses the *recA polA*-mediated lethality pathway^6,19^. We investigated the Srp pathway and identified the *srp* gene as *hslO*. Using an experimental synthetic lethality model of chromosome damage, we found that *recAts polA* cell growth arrest corresponded with elevated intracellular ROS levels at restricted temperatures. This growth arrest was ameliorated by eliminating hydrogen peroxide, which indicates that *recAts polA* lethality is reversible and at least partially mediated by ROS. It suggested that *recAts polA* lethality did not result from complete chromosome degradation which could be an irreversible incident. This raises the question of how *E. coli* cells maintain their chromosomes. The Srp pathway^3,19^ was originally observed in *recA200 polA25 lexA51* cells. These observations open the possibility that lethality suppression results from RecA200 protein renaturation via HslO chaperone activity in addition to *recA*-independent chromosome maintenance. In this study, we used a simplified genetic background where Srp was achieved by the *hslO* plasmid *p*EXsrpC. Also, we constructed *ΔrecA polA25* cells to eliminate the possibility of RecA200 renaturation. Our *ΔrecA polA25* cells had higher viability than with vectors alone. This result was in agreement with our previous results in *recAts polA25 Δsrp p*SrpC cells^6^. We also found that *ΔrecA polA25* cells show conditional growth arrest (or a lethal phenotype) when those cells were grown on the L medium. *ΔrecA polA25* cells with either an empty vector or *hslO*-expressing plasmid ameliorated L medium sensitivity with catalase. Thus, cells with complete loss of polymerase activity of DNA pol I and RecA activity do not completely lose their viability. This result suggests that DNA pol I, RecA, or their effectors are readily suppressed via alternative pathways in *recA polA* cells that are activated by chronic DNA damage.

The above conclusion opens another possibility for maintaining chromosome integrity. We have not yet elucidated how *ΔrecA polA25* cells maintain chromosomal integrity without *recA*. However, we show that cells can cope with chronic DNA damage from a loss of RecA function. In other words, intracellular ROS levels are another determinant for cell growth with DNA damage. In *recAts polA* cells, ROS accumulation and growth failure was observed at restricted temperatures. Catalase suppressed growth failure at restricted temperatures. Consistently, catalase also restored colony formation. These observations indicate that ROS accumulation was closely related to the growth failure of *recAts polA* cells. We must construct conditional phenomena to determine which events are required for cell survival. Thus, we investigated the conditions that cause cells to become inviable or fail to grow, because *ΔrecA polA25* cells are viable. We found that nutritional conditions are important for *recAts polA* cell viability and that growth failure is independent of heat shock. This suggests that cells require their chromosomes to be free from damage or that repair mechanisms are required for vigorous growth in the L medium. Further, our results highlight the possibility that ROS are common signals for close-knit cellular mechanisms. In our study, the associated cellular mechanisms were likely DNA damage sensing, DNA repair, chromosome replication, and cell division. HslO is likely involved in DNA damage sensing because *hslO* mutations sensitise cells to hypochlorite and hydrogen peroxide^8^. Chromosome breakage induces ROS production, so cells suffer ROS self-amplification. Our data suggest that *recA polA* lethality coincides with this possibility. First, we observed ROS accumulation only at restrictive conditions in *recA polA* cells. Second, ROS accumulation is titrated by the addition of catalase in those cells. Third, the ROS-related redox chaperone, *hslO*, can relieve ROS accumulation and growth defects in *ΔrecA polA25* cells. Thus, we conclude that the failure of *recA polA* cell growth partly results from ROS accumulation. Interestingly, it was proposed that a DNA checkpoint stops cell cycle progression to provide time to deal with DNA damage^20^. Thus, it is very likely that *hslO* expression protects cells from damage to gain sufficient time for repair when the cells produce detrimental levels of ROS.

It was noteworthy that only two cleavage sites were enough to cause ROS production and growth arrest. This indicates that DNA damage induces ROS production that is amplified and arrests cell growth. ROS-mediated cell regulation in *E. coli* is predicted by a ROS-mediated lethality mechanism^11^ or a redox-signalling pathway^21^, which is supported by this study. These findings suggest that HslO orchestrates cellular responses to high ROS levels. Therefore, HslO itself may serve as a deceleration device or protective molecule for chronic damage. In *recA polA* growth arrest or lethality, SOS responses are not induced due to *recA* deficiency^1^. Conversely, ROS production in damaged cells could be stimulated in these circumstances. Thus, DNA damage responses and regulation of cell division are likely involved in *recA polA* growth arrest or lethality. The expression of *hslO* might be regulated by RecA or LexA, as mentioned in our previous study^6^. Therefore, *hslO* will likely participate in later stages of DNA damage responses. In this study, ROS derived from metabolic processes were observed in the L medium. We do not know whether those metabolism-derived ROS and ROS caused by DNA damage are the same molecules. Recently, we started to evaluate highly reactive oxygen species (hROS)^22^ in *recA polA* cells. Hydrogen peroxide is converted into a highly reactive hydroxy radical. Thus, redox-signalling pathways are pivotal mechanisms underlying and regulating DNA metabolism in *E. coli*.

Redox molecular chaperones such as HslO play an extremely important role in oxidising conditions. These chaperones promptly detect oxidation stress, a possible cause of protein unfolding^23^. Once activated by oxidation, HslO protects proteins from becoming toxic^23,24^, which then protects bacterial cells from cell death. These studies focused on the role of oxidised HslO^7^. Restoring non-stress conditions reduces disulphide bonds in HslO, which then destabilises the bound substrate proteins and converts them into less structured proteins. This causes the folding of client proteins by ATP-dependent foldases^10^. Conversely, this may be another function of HslO to reduce ROS-triggered cellular responses. These unique characteristics of HslO are corroborated with our results. Coping with DNA damage stress leads to increased cell proliferation. Thus, redox-signalling pathways involving HslO are pivotal for understanding the underlying cellular mechanisms including DNA metabolism in *E. coli*.

## Methods

### *E. coli* strains and media

The *E. coli* strains used in this study are described in Table 1. Strains were constructed by phage P1*vir*-mediated transduction^25^. Cells were grown at 30 °C in M9 salt-glucose minimal (M9G) media^26^ supplemented with casamino acids (CAA) (0.2%; Difco Laboratories, Detroit, MI, USA), thymine (1 mg/mL), thiamine (1 μg/mL), appropriate amino acids (50 μg/mL): arg, thr, leu, trp, his, pro (M9GCAA medium), and antibiotics: ampicillin (20 μg/mL), kanamycin (55 μg/mL), spectinomycin (40 μg/mL), and streptomycin (100 μg/mL). Lennox broth was prepared as described by Miller (1992). The soft medium was prepared by adding 0.65% bactoagar.

**Table 1:** *Escherichia coli* strains used in this study.

Strain	Relevant genotype	Source, reference, or construct
AQ663	*ΔrecA306*	This laboratory
AQ8534	*polA25*::*spc zih-35*::Tn*10*	^33^
AQ10459	As AQ10458	^6^
AQ10549	*polA25 recA200*	^6^
AQ10870	AQ10459 *ΔrecA306*	^6^
AQ11471	*ΔhslO*::*Tc*	This study (Experimental procedure)
AQ11735	AQ10459 *polA25 zih-35*::Tn*10*	AQ10459 × P1.AQ8534 → Tc^r^, UV^s^
AQ11756	As AQ11735 but Tc^s^	Tc^s^ (Bochner selection)
TK1217	AQ11756 *p*MW119	AQ11756 × *p*MW119 → Ap^r^
TK1219	AQ11756 *p*EXsrpC	AQ11756 × pEXsrpC → Ap^r^
TK1224	TK1219 *ΔrecA306*	TK1219 × P1.AQ663 → Tc^r^
TK1230	TK1217 *ΔrecA306*	TK1217 × P1.AQ663 → Tc^r^
TK3078	AQ10549 *p*HSG576	AQ10549 × *p*HSG576 → Cm^r^
TK3079	AQ10549 *p*SRO1	AQ10549 × *p*SRO1 → Cm^r^
TK3080	AQ10549 *p*SRO*Δ*hslO	AQ10549 × pSRO*Δ*hslO → Cm^r^
TK3081	AQ10549 *p*SrpC	AQ10549 × pSrpC → Cm^r^
TK3082	AQ10549 *p*SRO*Δ*yrfG	AQ10549 × pSRO*Δ*yrfG → Cm^r^
TK3083	AQ10549 *p*SRO*Δ*hslRO	AQ10549 × pSRO*Δ*hslRO → Cm^r^

*Tr* temperature-resistant growth at 42 °C, *Ts* temperature-sensitive growth at 42 °C, *Tc*^*r*^*, Km*^*r*^*, Cm*^*r*^*, and Ap*^*r*^ denote resistance to tetracycline, kanamycin, chloramphenicol, and ampicillin, respectively, *Tc*^*s*^*, Km*^*s*^*, and Cm*^*s*^ denote susceptibility to tetracycline, kanamycin, and chloramphenicol, respectively, *UV*^*s*^ sensitive to UV, *UV*^*r*^ resistant to UV, *Tn* transposon.

AQ10458 genotype: *F-argE3 his-4 leuB-6 proA2 thr-1 thi-1 rpsL31 galK2 lacY1 mtl-1 supE44 sfiA11.*

### Cultivation and sampling methods

For cultivation, 2 and 15 mL of M9GCAA liquid medium were placed in test tubes and 100 mL Erlenmeyer flasks, respectively, inoculated with 1/100 volume of cells grown overnight in M9GCAA broth, and cultured aerobically at either 30 °C or 42 °C.

In shift-up experiments, cells were cultured in M9GCAA medium until OD_600_ = 0.1. Cells were then divided into two to four equal portions for reagent addition. After the indicated treatments, the cell cultures were measured for OD_600_, DNA content, and ROS analysis every 2 h from 0 to 16 h. For time-course experiments, typical sample volumes were 600 μL for OD_600_, 200 μL for DNA content, and 4 μL for ROS analysis.

For documenting their growth in the liquid medium, 10^7^ particles of TK1230 (*ΔrecA polA25 p*vec) or TK1224 (*ΔrecA polA25* pEX*srpC*) cells were inoculated into a mixed M9GCAA and L medium. TK1230 and TK1224 cells were cultivated for 30 h in a 2 mL liquid medium at 30 °C in test tubes. Moreover, 2 × 10^6^ TK1230 and TK1224 cell particles were mixed with 3 mL of either L or M9GCCA, poured onto soft agar plates, and cultivated for 3 days at 30 °C.

### Sequencing

DNA sequences were determined using a Sequenase Version 2.0 sequencing kit (USB Corp.). Analysis of the DNA sequences was performed using GCG sequence analysis software, version 3.0. Long PCR product sequences were determined as described previously^18^. The *hslO* gene sequence was retrieved from the KEGG database^27–29^ (url: https://www.genome.jp/kegg/kegg_ja.html), ecj: JW5692 or eco: b3401.

### qPCR analysis of target alleles

KAPA SYBR@FAST (Nippon Genetics, Tokyo, Japan) was used for qPCR. Chromosomal DNA was prepared using CellEase II bacteria kits (Cosmo Bio Co., LTD, Tokyo, Japan). The basic cycling parameters were as follows: primary denaturation of 94 °C for 3 min, followed by 40 cycles of 94 °C for 10 s, 56 °C for 10 s, and 72 °C for 10 s using a Light Cycler 96 (Roche, Basel, Switzerland). Primer sequences and targets included: polymerase domain of DNA pol I (*polA*) 5′-TTATCAAACGGGCGATGATT and 5′-GACGGGTACAGTTTTCCATCA; 5′–3′ exonuclease domain of DNA pol I 5′-CGGACGACGTTATCGGTACT and 5′-CACGCCGTACTTATTCACCA; and *recA* 5′-GGCCGTATCGTCGAAATCTA and 5′-ATATCGACGCCCAGTTTACG. Amplified DNA fragments were confirmed with ChIP electrophoresis using a Multina MCE-202 instrument with the DNA-500 reagent (Shimazu, Kyoto, Japan).

### Determination of cell survival and recovery

For relative viability (RV) determination, cells were incubated in an M9GCAA medium overnight at 30 °C. Then, the cells were diluted in an M9 medium without a nutrient source (M9B), plated on M9GCAA plates supplemented with appropriate antibiotics, and incubated for 16 h at either 30 °C or 42 °C. Then, 10^3^, 10^4^, and 10^5^ particles were spread on the plates. Cell viability was determined from the number of colonies grown on each plate.

In the complementation test, the RV was determined by comparing the number of colonies grown at a restrictive temperature with colonies at a permissive temperature.

In the spot method, 2 μL of diluted culture medium containing the indicated number of particles was smeared on the plate by spotting. The plates were incubated at 30 °C or 42 °C for 16 h. Grown cells on agar were collected from the surface of the spotted agar by wiping with a sterilised tip and were suspended in 20 μL of M9B.

For convenient streak analysis, 2 μL of cells from an overnight culture were spotted on Lennox agar (LA) plates and were spread with a sterilised toothpick. Then, 3 mL of soft LA was overlaid on the plates and incubated at either 30 °C or 42 °C.

For the colony formation assay, 2 × 10^6^ cells from an overnight culture were added to 3 mL of soft M9GCAA or LA medium and were poured on M9GCAA or LA plates. The plates were incubated at 30 °C or 42 °C.

### Plasmid construction

The 3.4 kb DNA fragment of *srp*, a suppressor of *recA polA* lethality^6^, was cloned into the BamHI site on *p*HSG576^30^. The resultant plasmids were termed *p*SRO1. *p*SRO1, *p*SRO*ΔhslO,* and *p*SrpC were described previously^6^. To construct *p*SRO*ΔyrfG*, *p*AQ10917 was digested with NsiI and BsmBI and self-ligated. In self-ligations, 1 μg digested DNA was blunted using 1 unit of T4 DNA polymerase (New England Bio Labs, Ipswich, MA, USA) supplemented with 1 mM dNTPS in NEBbuffer 1.1 at 12 °C for 20 min in 20 μL reaction, followed with heat inactivation at 75 °C for 30 min. Ethanol-precipitated blunted DNA was then ligated with 250 units of T4 DNA ligase (Nippon gene, Tokyo, Japan) in manufacturer-supplemented reaction buffer at 16 °C for 12 h. Then, a BamHI DNA fragment with a *yrfG* deletion was cloned into pHSG576. Subsequently, an *spc* cassette was cloned into the EcoRI site. The resultant plasmid was called *p*SRO*ΔyrfG*. To construct *p*SRO*ΔhslRO*, *p*AQ10917 was digested with BsmBI and BstEII and self-ligated. Subsequently, a BamHI DNA fragment containing a *hslR to hslO* deletion was cloned into *p*HSG576. Then, an *spc* cassette was cloned into the EcoRI site. The resultant plasmid was called *p*SRO*ΔhslRO*.

### Flow cytometry

For CellRox Deep Red analysis using flow cytometry^31^, staining was performed according to our previous study^6^. Cell cultures (4 μL) at the indicated times were mixed with 12.5 μM CellRox Deep Red (16 μL), diluted with M9 medium without organic nutrients (M9B), and incubated for 30 min at 25 °C. Stained cells (20 μL) were then diluted in M9B (200 μL). We then used a Becton Dickinson Accuri C6 (Becton, Dickinson and Company, Ann Arbor, MI, USA) flow cytometer equipped with a 640 nm laser. First, we analysed cells to set the gating scheme. We used identical side scatter signal/forward scatter signal (FSC) gates (designated as P3) to collect 50,000 events. In our experiments, the event rate was less than 2500 events per second. The data were analysed with C6 software, version 1.0.264.21. Each sample was plotted as a histogram vs. the red channel (FL4-A with 675 ± 15 nm filter), ROS content (fluorescence, channel FL4-A), autofluorescence from the green channel (FL-1A), or as a function of cell size (determined by FSC).

For double staining, we used a P4 gate. CellRox Deep Red staining was performed as described above, and SYBR Green I staining was performed by adding M9B (16 μL) containing 1.25 × SYBR Green I to the bacterial recovery solution (4 μL) for 30 min. To analyse *E. coli* particles with nucleic acids, unstained and SYBR Green I stained cells were compared. M-1 was set as the gate for nucleic acids. The leakage of unstained particles into the M-1 channel was less than 0.1%. The amount of ROS was analysed based on the FL-4A channel, which detected particles with nucleic acids. DNA content analysis was carried out as described previously^6,32^.

A BD Cell Viability Kit (Becton, Dickinson and Company, 335925) was used to determine the number of particles in the cultures.

### Statistical analysis

The data are represented as means ± standard error of the mean (SEM), which were calculated with the STDEV.P function in Microsoft Excel Plus 2019. Welch’s *t* test in Excel was used to analyse statistical differences, with *p* < 0.05 or *p* < 0.01.

## Supplementary Information

Supplementary Figures.

## Data Availability

The data that support the findings of this study are available from the corresponding author upon reasonable request. Our long PCR product sequences have been deposited to DDBJ as follows. The fastq data obtained from AQ10459, TK1224, TK1230 and AQ10870, respectively, were registered in the DDBJ Sequence Read Archive (DRR417949, DRR417950, DRR417951, DRR417952). AQ10459 (DRR417949): https://ddbj.nig.ac.jp/resource/sra-run/DRR417949. TK1224 (DRR417950): https://ddbj.nig.ac.jp/resource/sra-run/DRR417950. TK1230 (DRR417951): https://ddbj.nig.ac.jp/resource/sra-run/DRR417951. AQ10870 (DRR417952): https://ddbj.nig.ac.jp/resource/sra-run/DRR417952. The nucleotide sequences of strains AQ10459, TK1224, TK1230 and AQ10870 were submitted to GenBank under accession numbers LC733663, LC733664, LC733665 and LC733666 respectively. AQ10459 (LC733663): http://getentry.ddbj.nig.ac.jp/getentry/na/LC733663?filetype=html. TK1224 (LC733664): http://getentry.ddbj.nig.ac.jp/getentry/na/LC733664?filetype=html. TK1230 (LC733665): http://getentry.ddbj.nig.ac.jp/getentry/na/LC733665?filetype=html. AQ10870 (LC733666): http://getentry.ddbj.nig.ac.jp/getentry/na/LC733666?filetype=html.
