# Supplementary material for: HslO ameliorates arrested ΔrecA polA cell growth and reduces DNA damage and oxidative stress responses
Source: Sci Rep. 2022 Dec 23;12:22182. doi: 10.1038/s41598-022-26703-z (PMC9789031; doi:10.1038/s41598-022-26703-z)
Supplement: Supplementary file 1 — Supplementary Figures. [file 41598_2022_26703_MOESM1_ESM.docx]

**Supplementary Information**

**HslO ameliorates arrested *ΔrecA polA* cell growth and reduces DNA damage and oxidative stress responses**

A. Kaidow*^1, 3^, N. Ishii^1^, S. Suzuki^2^, T. Shiina^2^, K. Endoh^1^, Y. Murakami^1^ and H. Kasahara^1^

^1^Department of Biology, School of Biological Sciences, Tokai University, Sapporo 005-8601, Japan

^2^Department of Molecular Medicine, School of Medicine, Tokai University,

Isehara 259-1193, Japan

^3^Hokkaido Regional Research Center, Tokai University, Sapporo 005-8601, Japan

**^*^Corresponding author:**

Akihiro Kaidow

Department of Biology, School of Biological Sciences, Tokai University, Sapporo 005-8601, Japan

Phone: 81-11- 571-5111 (ex2912)

Fax: 81-11-571-7879

E-mail: akaidow@tsc.u-tokai.ac.jp

**Supplementary Figures**

**
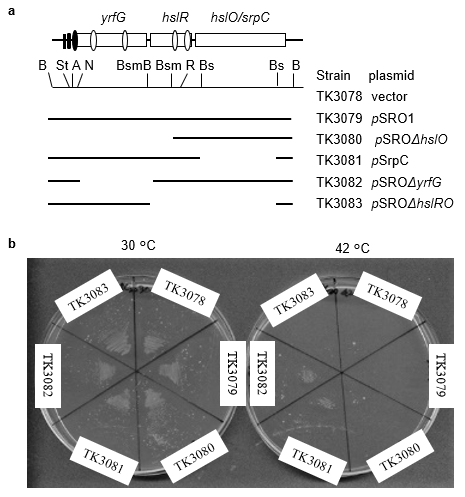
**

**Fig. S1 Suppression of temperature sensitivity of *recAts polA* cells with *hslO* plasmids**

**(a) Schematic representation of the gene organisation of the *srp* operon**

*srp* operon genes are indicated as open boxes. Black boxes upstream of *yrfG* indicate LexA-like sequences. Promoters in the *srp* region are shown as ovals. The restriction endonuclease cleavage sites were as follows: B, BamHI; P, PflMI; X, XmnI; St, StuI; A, AflIII; N, NsiI; E, EcoRV; Ag, AgeI; Sp, SphI; BsmB, BsmBI; Bsm, BsmI; R, RsrII; Bs, BstEII; and S, SalI. DNA fragments harboured in individual plasmids are represented by bars.

**(b) Suppression of temperature sensitivity in AQ10549 cells harbouring different fragments of the *srp* operon**

Overnight cultures (2 × 10^7^ cells) of the indicated cells were spotted in each sector and streaked as single colony isolation (S.C.I.) with a toothpick. Then, plates were overlaid with 3 mL soft LA medium. Plates were incubated at either 30°C or 42°C for 2 days. Cells harbouring plasmids are as described in (a).

**
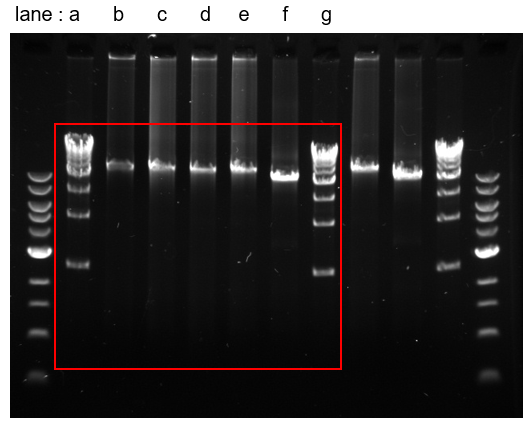
**

**Fig. S2 Original gel electrophoresis photograph of the long PCR shown in Fig. 1b**

Long PCR was carried out as described in the Materials and Methods. The results of 0.8% agarose gel electrophoresis are shown. Lane a: marker, lane b: TK1224 cells, lane c: TK1230 cells, lane d: *ΔrecA306* derivative, lane e: AQ10870 cells, lane f: AQ10459 cells, lane g: marker. The gel image in the red box was flipped and then presented in Fig. 1b. Thus, lanes a to g in Fig. S2 correspond to lanes 7 to 1 in Fig. 1b, respectively.

**
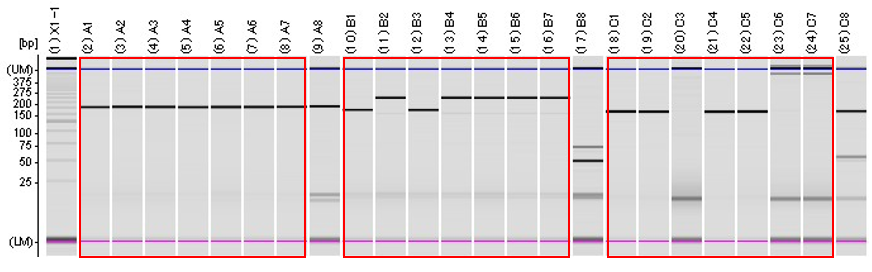
**

**Fig. S3 The original gel image of the qPCR shown in Fig. 2b, c, d**

The qPCR products were analysed using ChIP electrophoresis. The original gel image was created automatically by Multina-202. The three red boxes from left to right correspond to Fig. 2b, c, and d, respectively. Lanes A1 to A7: 5′-3′ exonuclease, Lanes B1 to B7: polymerase, and Lanes C1 to C7: *recA* fragments.

**
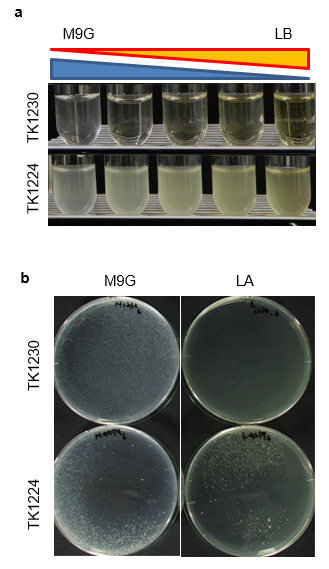
**

**Fig. S4 Rich plate medium sensitivity of *ΔrecA306* *polA25* cells**

**a) L broth medium sensitivity of *ΔrecA306* *polA25* cells**

Fully grown TK1230 or TK1224 cells were inoculated in various mediums at 2 × 10^7^ particles/mL (approximately 0.01 OD_600_), and were cultured for 30 h at 30°C. From left to right: M9GCCA, 25% L medium with M9GCCA, 50% L medium with M9GCCA, 75% L medium with M9GCCA, and 100% L medium.

**b) L plate medium sensitivity of *ΔrecA306* *polA25* cells**

Fully grown TK1230 or TK1224 cells were diluted with M9B. Then, 10^6^ particles of TK1230 or TK1224 cells were mixed with either soft MPGCCA medium or soft LA medium and were poured on M9GCCA or LA plates. Plates were cultured for 3 d at 30°C.
